# Divergent Dimethylarginine Dimethylaminohydrolase Isoenzyme Expression in the Central Nervous System

**DOI:** 10.1007/s10571-021-01101-7

**Published:** 2021-05-20

**Authors:** Alena A. Kozlova, Vinitha N. Ragavan, Natalia Jarzebska, Iana V. Lukianova, Anastasia E. Bikmurzina, Elena Rubets, Toshiko Suzuki-Yamamoto, Masumi Kimoto, Arduino A. Mangoni, Raul R. Gainetdinov, Norbert Weiss, Michael Bauer, Alexander G. Markov, Roman N. Rodionov, Nadine Bernhardt

**Affiliations:** 1 Department of Psychiatry and Psychotherapy, University Hospital Carl Gustav Carus, Technische Universität Dresden, Dresden, Germany; 2 Institute of Translational Biomedicine and Saint-Petersburg University Hospital, Saint-Petersburg State University, Saint-Petersburg, Russia; 3 University Centre for Vascular Medicine and Department of Internal Medicine, Technische Universität Dresden, Dresden, Germany; 4 Department of Clinical Pharmacology, College of Medicine and Public Health, Flinders University and Flinders Medical Centre, Adelaide, Australia; 5 Department of Anesthesiology and Intensive Care Medicine, University Hospital Cart Gustav Carus, Technische Universität Dresden, Dresden, Germany; 6 Department of General Physiology, Saint-Petersburg State University, 199034 Saint-Petersburg, Russia; 7 Department of Nutritional Science, Faculty of Health and Welfare Science, Okayama Prefectural University, Okayama, Japan

**Keywords:** DDAH1, DDAH2, Brain, Mouse, Human

## Abstract

The endogenous methylated derivative of ʟ-arginine, *N*^*ω*^,*N*^*ω*′^-dimethyl-ʟ-arginine (asymmetric dimethylarginine, ADMA), an independent risk factor in many diseases, inhibits the activity of nitric oxide synthases and, consequently, modulates the availability of nitric oxide. While most studies on the biological role of ADMA have focused on endothelial and inducible nitric oxide synthases modulation and its contribution to cardiovascular, metabolic, and renal diseases, a role in regulating neuronal nitric oxide synthases and pathologies of the central nervous system is less understood. The two isoforms of dimethylarginine dimethylaminohydrolase (DDAH), DDAH1 and DDAH2, are thought to be the main enzymes responsible for ADMA catabolism. A current impediment is limited knowledge on specific tissue and cellular distribution of DDAH enzymes within the brain. In this study, we provide a detailed characterization of the regional and cellular distribution of DDAH1 and DDAH2 proteins in the adult murine and human brain. Immunohistochemical analysis showed a wide distribution of DDAH1, mapping to multiple cell types, while DDAH2 was detected in a limited number of brain regions and exclusively in neurons. Our results provide key information for the investigation of the pathophysiological roles of the ADMA/DDAH system in neuropsychiatric diseases and pave the way for the development of novel selective therapeutic approaches.

**Supplementary Information:**

The online version contains supplementary material available at 10.1007/s10571-021-01101-7.

## Introduction

Dimethylarginine dimethylaminohydrolases (DDAH) are a family of enzymes that metabolize methylated arginines. In mammals, there are two isoforms, DDAH1 and DDAH2, which have around 50% identity in their amino acid sequences (Frey et al. 2006) and differential tissue expression patterns (Leiper et al. 1999; Dayal et al. 2008). *DDAH1* mRNA is widely expressed in mammals, particularly in the kidney, brain, and liver, while *DDAH2* mRNA is primarily found in the heart, lung, and placenta (Dayal et al. 2008; Leiper et al. 1999). DDAH1 was first discovered by Ogawa and colleagues, who also identified that its catabolic reaction results in the formation of citrulline and dimethylamine (Ogawa et al. 1987). There are four forms of methylated arginine, which are formed ubiquitously through degradation of proteins (Kakimoto and Akazawa 1970; McDermott 1976), namely *N*^*ω*^-monomethyl-ʟ-arginine (ʟ-NMMA), *N*^*ω*^,*N*^*ω*′^-dimethyl-ʟ-arginine (asymmetric dimethylarginine, ADMA), *N*^*ω*^,*N*^*ω*^-dimethyl-ʟ-arginine (symmetric dimethylarginine, SDMA), and *N*-δ-methylarginine, which is found in yeast and human cells (Niewmierzycka and Clarke 1999; Zobel-Thropp et al. 1998; Martens-Lobenhoffer et al. 2016). However, in mammals, only ʟ-NMMA and ADMA are primarily hydrolyzed by the DDAH enzymes, while SDMA is mainly removed by renal excretion (Ogawa et al. 1989; McDermott 1976; Leiper et al. 1999).

Research on DDAH has mostly focused on its role in metabolizing ADMA, given that plasma concentrations of ADMA are higher than those of ʟ-NMMA (Meyer et al. 1997). Moreover, ADMA is linked to various pathologies; with elevated ADMA concentration observed in endothelial dysfunction, chronic renal failure, hypertension, heart failure, diabetes, and atherosclerosis (Boger 2005; Zoccali et al. 2001; Usui et al. 1998; Miyazaki et al. 1999; Leone et al. 1992; Vallance et al. 1992; Chen et al. 2012). ADMA is a potent inhibitor of nitric oxide synthases (NOSs), a family of enzymes responsible for the production of nitric oxide (NO). Thus, DDAH inhibition leads to increased ADMA concentrations and, consequently, reduced NO availability (MacAllister et al. 1996). Conversely, overexpression of DDAH1 results in reduced ADMA concentration and increased NO production (Ayling et al. 2006). Furthermore, ADMA causes uncoupling of NOS, a process that leads to the production of superoxides (Toth et al. 2007; Antoniades et al. 2009).

NO is a gaseous signaling molecule that is involved in various biological processes, including the modulation of central nervous system (CNS) functions (Calabrese et al. 2007). There are three variants of the NOS enzymes, neuronal NOS (nNOS), inducible NOS (iNOS) and endothelial NOS (eNOS). Within the CNS, nNOS and eNOS have been shown to regulate learning and memory formation, neurogenesis, and long-term synaptic transmission, while iNOS has an immunoregulatory function (Asif et al. 2013; Chen et al. 2005; Böhme et al. 1993; Hölscher et al. 1996; Son et al. 1996; Zhou et al. 2007; Sonar and Lal 2019). Upregulation of nNOS and/or iNOS has been implicated in brain ischemia and the pathogenesis of neurodegenerative diseases such as Parkinson’s disease and Alzheimer’s disease (Zhou et al. 2007; Gatto et al. 2000; Izumi et al. 1992; Gang Zhang et al. 1994; Hannemann et al. 2020). Numerous studies have also demonstrated the key role of the nNOS-NO pathway in the etiology of affective disorders such as depression (Selley 2004; Baranyi et al. 2015; Ozden et al. 2020) and bipolar affective disorder (Sağlam Aykut et al. 2012; Ustundag et al. 2020). This suggests that the modulation of NOS activity may have therapeutic effects also in CNS disease states (reviewed in Dhir and Kulkarni 2011; Freudenberg et al. 2015). As ADMA is the major endogenous inhibitor of NOS, targeting DDAH may represent a promising strategy for the development of new treatment approaches.

The function of DDAH isoforms in the brain however, remains elusive. Assessing the pathophysiological role of DDAH in the brain requires in-depth knowledge of regional and cellular DDAH expression profiles to inform how modulation of DDAH will affect different brain regions or cellular subtypes. We sought to address this issue by providing detailed mapping of DDAH1 and DDAH2 regional and cellular protein expression in the murine brain and comparative analysis in human post-mortem tissue samples.

## Materials and Methods

### Animals and Tissue Collection

Experiments were conducted in ten male C57Bl/6 J mice 10–12 weeks old purchased from the Jackson Laboratory, USA. Animals were housed in a 12-h light–dark cycle (lights on at 06:00) with food and water ad libitum. All efforts were made to reduce animal suffering and the number of animals used. Mice were deeply anesthetized with a mixture of 100 mg/kg ketamine and 10 mg/kg xylazine, transcardially perfused with phosphate buffered saline (PBS) followed by 4% paraformaldehyde (PFA). Brains were dissected and post-fixed in 4% PFA overnight, cryoprotected in 20% sucrose in PBS for up to 4 days, frozen in methylbutane at − 40 °C and then stored at − 80 °C. Coronal or sagittal sections of 40 µm thickness were cut on a freezing microtome (Leica CM1850) and stored in antifreeze (25% glycerol, 25% ethylene glycol in PBS) at − 20 °C until further processing.

### Human Tissue Collection

Frontal gyrus samples from normal human brain tissue were obtained through the National Health and Medical Research Council South Australian Brain Bank. Brain samples were extracted and embedded in paraffin. Sample blocks were cut in 8 µm sections and mounted onto gelatin-coated slides. Additional brain tissue samples were obtained from a 49-year-old man diagnosed with acute lymphoblastic leukemia obtained from N.N. Petrov National Medical Research Center of Oncology, Russia. Brain samples were extracted and dissected according to The Human Brain Atlas into the prefrontal cortex and amygdala (The Human Brain 2020). Samples were post-fixed in 10% PFA for seven days and then cryoprotected in 15% sucrose in PBS for up to 4 days. Sections of 35 µm thickness were cut on a freezing microtome (Leica CM1850UV) and placed directly on glass slides.

### Immunohistochemical Staining

Brain sections were deparaffinized by incubation in xylene followed by washing with decreasing ethanol concentrations (100%, 95%, 75%) for 5 min each. All further washing steps were performed in tris-buffered saline (TBS) with azide (TBS with 0.1% sodium azide). For elimination of endogenous peroxidase activity, slides were covered with 1% H_2_O_2_–50% methanol and placed in a humidified chamber for 10 min. Then, sections were washed and blocked in 20% normal horse serum (NHS) in TBS-azide for 1 h. Slides were incubated overnight with primary antibody solution (1% NHS in TBS-azide). All antibodies and respective dilutions used are listed in Table 1. Sections were washed 3 × 5 min and incubated with biotin-conjugated secondary antibody solution (1% NHS in TBS-azide). Sections were further washed 3 × 5 min and incubated with ABC-kit solution (Vector PK-4000, Vectastain) for 60 min. Sections were again washed 3 × 5 min and exposed to the DAB reaction (D4293, SigmaFast, dissolved in 5 ml of TBS and 3.5 µl H_2_O_2_). The reaction was stopped with TBS-azide. After brief washing, sections were counter-stained with hematoxylin, dehydrated (three washes in 100% ethanol, two washes in xylene) and mounted with DPX (mixture of distyrene, a plasticizer, and xylene).

**Table 1:** List of used antibodies

Primary antibodies	Hosts	Dilution	Identifier
Anti-DDAH1	Rabbit	1:500	PA5-52278 (ThermoFisher)
Anti-DDAH1	Mouse	1:500	clone 3H10 (Prof. Masumi Kimoto, Okayama Prefectural University)
Anti-DDAH1	Rabbit	1:500	HPA006308 (MERCK)
Anti-DDAH1	Goat	1:10000	ab2231 (Abcam)
Anti-DDAH2	Rabbit	1:200	ab232694 (Abcam)
Anti-DDAH2	Goat	1:1000	ab1383 (Abcam)
Anti-DDAH2	Rabbit	1:1000	STJ28540 (St. John’s)
Anti-GFAP	Goat	1:1000	ab53554 (Abcam)
Anti-NeuN	Mouse	1:1000	ab104224 (Abcam)
Anti-Olig2	Rabbit	1:250	ab109186 (Abcam)
Anti-Iba1	Rabbit	1:1000	019-19741 (Wako Chemicals)
Anti-CD31	Mouse	1:500	ab64543 (Abcam)
Anti-S100	Mouse	1:200	ab868 (Abcam)

Secondary antibodies	Host	Dilution	Company
Anti-rabbit Alexa 488	Goat	1:1000	ab150117 (Abcam)
Anti-rabbit Alexa 648	Donkey	1:1000	ab150067 (Abcam)
Anti-mouse Alexa 568	Goat	1:1000	ab175701 (Abcam)
Anti-goat Alexa 488	Donkey	1:1000	ab150133 (Abcam)

### Immunofluorescence

Immunofluorescence staining on mouse samples was carried out on free-floating sections. For human samples, antigen retrieval was performed by boiling the slides in citrate buffer (0.01 M, pH 6.0) for 10 min. Initial washing was performed 3 × 15 min in PBS followed by blocking in 10% normal donkey serum (NDS, ab7475, Abcam) and 0.2% Triton X-100 in PBS for 2 h at room temperature. Sections were then incubated overnight at 4 °C in primary antibody solution (3% NDS and 0.2% Triton X-100 in PBS). Sections were washed 3 × 15 min with PBS and incubated with fluorescence conjugated secondary antibody solution (3% NDS and 0.2% Triton X-100 in PBS) for 2 h at room temperature. All antibodies tested are listed in Supplementary Tables S1 and S2, while the antibodies used for the final study, including the dilutions, are listed in Table 1. Sections were then washed 3 × 15 min in PBS and nuclei were counterstained for 10 min with 4′,6-diamidino-2-phenylindole (DAPI) in PBS. After a final 10 min wash, sections were mounted on glass slides (SuperFrost Ultra Plus, Thermo Scientific) with Mowiol (MERCK, #475,904). For double staining, sections were incubated in mixed primary antibody solutions. Samples were visualized and analyzed using Zeiss Observer Z.1 (ApoTome II) widefield microscopy or Zeiss LSM880 confocal microscopy, using Zen 3.2 (blue edition) and ImageJ 1.50e software (Schneider et al. 2012).

## Results

Comprehensive antibody validation experiments were carried out using western blot and cell and tissue immunostaining. This approach allowed analysis of various available antibodies against DDAH1 and DDAH2 in order to identify the most specific antibodies for the final study (Supplementary Tables S1 and S2, Supplementary Material Figs. S1 and S2). We focused on the molecular mass of the protein band and its intensity in addition to the specificity of antibody binding with endogenous DDAH1 and DDAH2.

### DDAH1 Distribution in the Mouse Brain

**Fig. 1:**
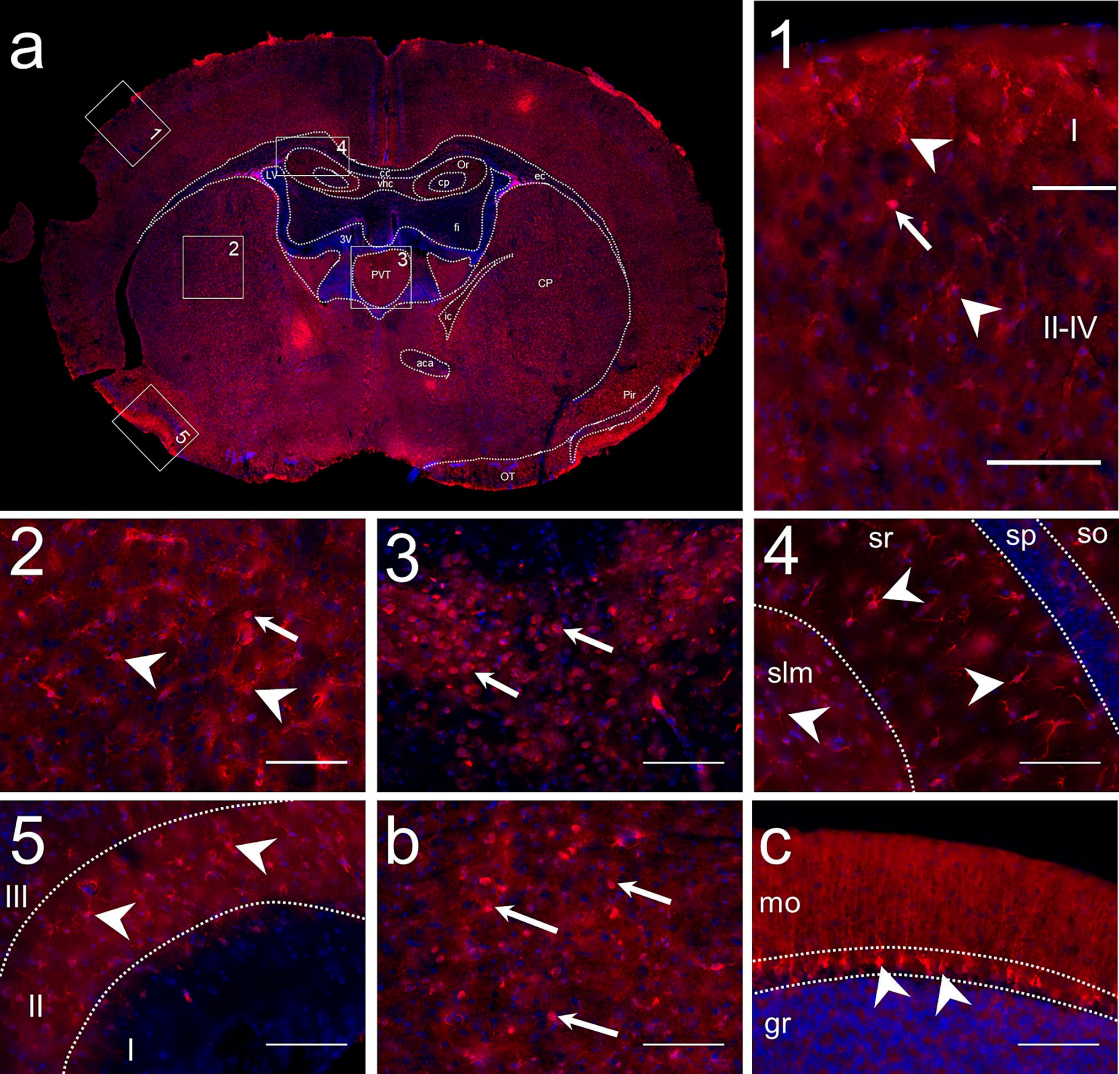
DDAH1 is widely distributed in the mouse brain. To assess patterns of DDAH1 protein expression, coronal mouse brain sections were stained with anti-DDAH1 antibody. **a** Representative section from Bregma -0.94. Regions with intense DDAH1 expression include: 1. DDAH1 positive cells in the cortex in all layers I and II-IV; 2. Intense DDAH1 expression in caudoputamen (CP); 3. DDAH1 positive cells in the paraventricular nucleus of the thalamus (PVT) appear in a round shape; 4. DDAH1 cells are present in HPF, in stratum oriens (so), stratum radialis (sr) and in stratum lacunosum-moleculare (slm), but not in pyramidal layer (sp); 5. DDAH1 immunoreactivity in the layer II in the piriform cortex. **b** DDAH1 positive cells in dorsal raphe. **c** DDAH1 positive cells specific to Purkinje layer of cerebellum; mo—molecular layer, gr—granular layer. Nuclear staining in blue with DAPI. White arrows and arrowheads indicate DDAH1 immunoreactive cells with differing morphology. Scale bars 100 µm

To investigate DDAH1 protein distribution in the brain, we initially used anti-DDAH1 staining on mouse brain sections. As shown in Fig. 1a, DDAH1 was widely distributed with an intense signal in the striatum, cortex and hippocampal formation (HPF). A moderate signal was observed in the thalamus and cerebellum (CB). In the striatal regions, cortex, HPF and CB the DDAH1 positive cells had an area of 12–16 µm^2^, star-shaped soma and many processes. In addition, a few cells of the same size but with a round soma and low expression level of DDAH1 were observed. Moreover, DDAH1 positive cells with star-shaped morphology were detected in white matter structures such as corpus callosum. In the thalamus and in some structures of the midbrain and hindbrain, low levels of DDAH1 expression were observed in round-shaped cells with 16–18 µm^2^ area, which was slightly bigger than the cells in the HPF and striatum.

Within cortical regions, DDAH1 immunoreactivity was intense in layers I–IV and isocortex (Fig. 1a, box 1). In the striatum, cells with DDAH1 expression were evenly distributed. DDAH1 positive cells were detected in both the dorsal and ventral regions, in the lateral septal complex, and, in a small amount, in the striatum-like amygdalar nuclei. The most intense DDAH1 expression, however, was observed in the dorsal region of the striatum (Fig. 1a, box 2). In the thalamus, the DDAH1 signal was moderate and present only in a limited number of brain nuclei such as the paraventricular nucleus (PVT) (Fig. 1a, box 3) and the reticular nucleus (RT). In HPF, few DDAH1 positive cells were present in Ammon’s horn (cornu Ammonis, CA) and the dentate gyrus (DG). In the CA, DDAH1 positive cells were observed in the stratum oriens (so), stratum radialis (sr), and stratum lacunosum-moleculare (slm) layers (Fig. 1a, box 4). In the DG, strong DDAH1 expression was present in the polymorph and molecular layers. Interestingly, no DDAH1 positive cells were found in both the pyramidal layer (sp) of CA and the granule cell layer in the DG. However, we observed some processes of DDAH1 positive cells that were passing through these layers. DDAH1 immunoreactivity was strong in different olfactory areas. High expression levels were observed in the main olfactory bulb (MOB), anterior olfactory nucleus (AON) and in the layer II of the piriform cortex (Fig, 1a, box 5). In CB, DDAH1 immunoreactivity was low in the Purkinje layer, without any detectable DDAH1 expression in other layers and cerebellar nuclei (Fig. 1c). Additionally, some DDAH1 positive cells were observed in the dorsal nucleus Raphe (Fig. 1b).

We compared our findings to publicly available mRNA expression datasets (Allen Institute for Brain Science 2010; Linnarsson Lab 2020), observing similar and intense expression profiles in the cortex, CB and MOB as well as low expression in the hypothalamus. Further high levels of DDAH1 protein in the striatum and HPF mapped to low *Ddah1* expression (Allen Institute for Brain Science 2010). On the contrary, low IHC reactivity reflected intense *Ddah1* expression in the ventral tegmental area. Notably, the strongest *Ddah1* mRNA signals were found in a wide number of thalamic nuclei (Allen Institute for Brain Science 2010), while we observed only moderate DDAH1 protein expression restricted to a small number of nuclei, including the PVT and RT (see Supplementary Table S3).

### DDAH1 is Expressed in Both Neuronal and Glial Cells

**Fig. 2:**
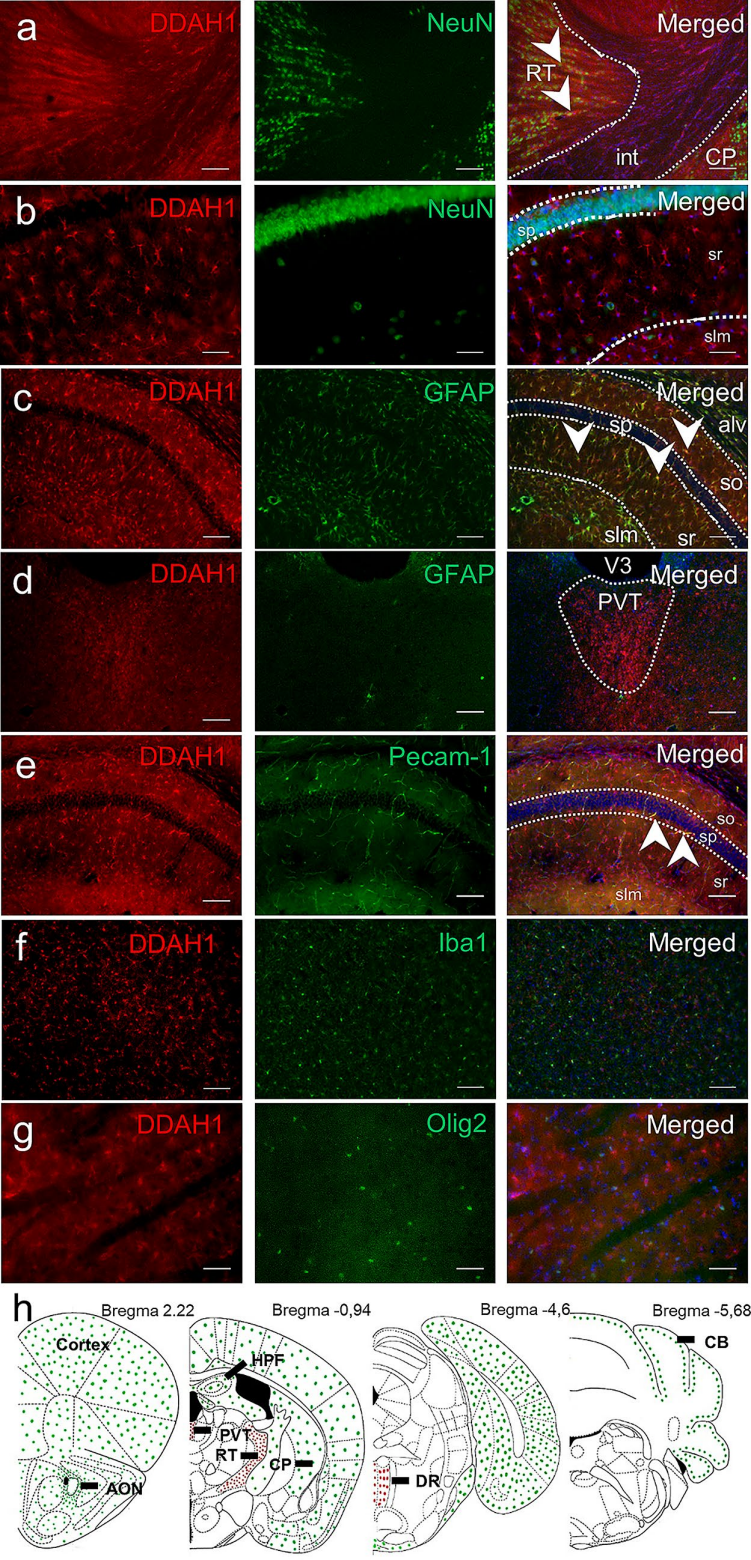
DDAH1 expression in different cell types. Mouse brain sections were stained with anti-DDAH1 antibody (red, first column) and main cell markers: neuronal marker (NeuN), glial fibrillary acidic protein (GFAP), oligodendrocyte transcription factor (Olig2), ionized calcium-binding adapter molecule 1 (Iba1), platelet/endothelial cell adhesion molecule 1 (PECAM-1), (green, second column). **a** Co-staining DDAH1 and NeuN in the RT; int – internal capsule. **b** DDAH1 signal in the HPF did not overlap with NeuN but showed complete overlap with GFAP in all hippocampal layers and with some cells in white matter of alveus **c**, **d** No overlap of staining of DDAH1 and GFAP in PVT. **e** Co-labeling of DDAH1 and PECAM-1 is observed in vessels in the HPF. **f** Striatal example of staining with DDAH1 and Iba1, no overlay between these proteins was observed. **g** Similarly, no overlap between DDAH1 and Olig2 was observed. **h** Overview scheme summarizing DDAH1/NeuN positive cells (red dots) and DDAH1/GFAP positive cells (green dots) and their correspondence to brain structures: cortex, anterior olfactory nucleus (AON); caudoputamen (CP); hippocampal formation (HPF); reticular nucleus of the thalamus (RT); paraventricular nucleus of the thalamus (PVT); dorsal Raphe (DR); cerebellum (CB); stratum oriens (so); stratum radialis (sr); stratum lacunosum-moleculare (slm); single cells in pyramidal layer (sp); alveus (alv); 3^d^ ventricle (V3). Nuclei staining in blue with DAPI. White arrows mark overlaps between cell marker (green) and DDAH1 + cells (red). Scale bar is 100 µm

Next, we determined specific cell type expression by co-labeling analysis of DDAH1 with a neuronal marker (NeuN), astrocyte markers glial fibrillary acidic protein (GFAP) and S100 calcium-binding protein B (S100beta), oligodendrocyte transcription factor (Olig2), ionized calcium-binding adapter molecule 1 (Iba1) and platelet/endothelial cell adhesion molecule 1 (PECAM-1) (Fig. 2h)**.** On the one hand, we found an overlapping signal between the neuronal marker NeuN and DDAH1 in RT and PVT (Fig. 2a) but no overlap between DDAH1 and the astrocyte marker GFAP (Fig. 2d). On the other hand, DDAH1 and GFAP were expressed by the same cells in all layers of the HPF (Fig. 2c), but we did not observe any overlap between DDAH1 and NeuN (Fig. 2b). In cortical and striatal regions, DDAH1 cells did overlap with S100beta (Supplementary Material, Fig. S3). Immunocytochemical analysis on primary cell culture of cortical and hippocampal origin confirmed the presence of DDAH1 in astrocytes and neurons (Supplementary Material, Fig. S4a-c). Additionally, we performed co-staining for DDAH1 and other cell-type markers such as Olig2 for oligodendrocytes, Iba1 for microglia and PECAM-1 for endothelial cells. We observed partial co-labeling between DDAH1 and PECAM-1 throughout the brain, however the majority of PECAM-1 did not overlap with DDAH1 (Fig. 2e). Despite prevalent Iba1 expression, e.g., within the striatum, we did not detect any co-labeling with DDAH1 (Fig. 2f). Furthermore, no co-labeling between DDAH1 and Olig2 was found (Fig. 2g). In summary, we found that DDAH1 is widely distributed in the rodent brain and expressed in a region-specific manner in both neuronal and astrocyte cells as well as within the endothelium of the vascular structures.

### DDAH2 Protein is Expressed in a Limited Number of Brain Regions

**Fig. 3:**
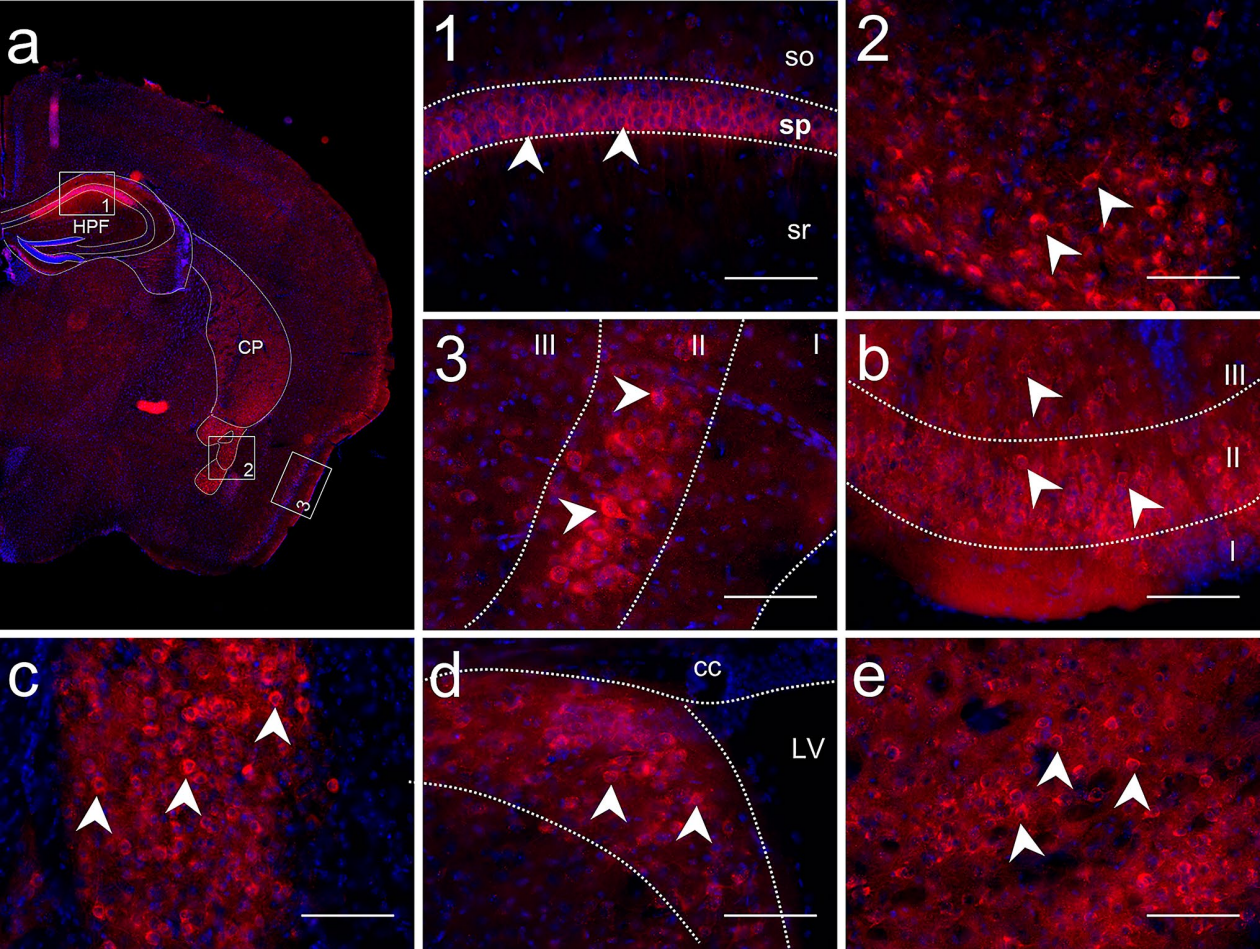
Immunohistological analysis of mouse brain sections revealed a limited number of regions with DDAH2 expression. To assess patterns of DDAH2 expression, mouse brain sections were stained with anti-DDAH2 antibody. **a** Overview of coronal sections with regions of intense DDAH2 expression: 1. DDAH2 positive cells in pyramidal layer (sp) of CA1 in the hippocampal formation but not in stratum oriens (so), stratum radialis (sr); 2. DDAH2 positive cells in the central amygdalar nuclei (CEA); 3. DDAH2 immunoreactivity in the layer II in the piriform cortex. **b** DDAH2 expression in the II and III layer of the olfactory tubercule. **c** DDAH2 positive cells in the bed nuclei of the stria terminalis. **d** DDAH2 positive cells in the lateral septal nucleus; LV—lateral ventricle, cc—corpus callosum. **e** Intense DDAH2 expression in the fundus of the striatum. Nuclear staining in blue with DAPI. White arrows indicate DDAH2 immunoreactivity. Scale bar is 100 µm

To build a map of DDAH2 protein expression, we firstly investigated its distribution on consecutive coronal mouse brain sections. As shown in Fig. 3a, DDAH2 immunoreactivity was detected in a limited number of brain structures. We found high expression levels in the cortex, HPF, striatum, and pallidum. Low expression was observed in the cortical subplate. In all structures, DDAH2 positive cells had a round-shaped soma, an area of 15–19 µm^2^ and stained processes.

Within the striatum, intense DDAH2 staining was observed in the ventral region, the lateral septal complex and the striatum-like amygdalar nuclei but not in the dorsal part. DDAH2 positive cells were observed in the lateral septal nucleus (LSN) (Fig. 3d) of the lateral septal complex. Here, DDAH2 signal was detected in the caudal part of LSN, while no staining was apparent in the rostral and ventral parts. In the ventral striatum, DDAH2 positive cells were found in the fundus of the striatum (FS) (Fig. 3e) and the olfactory tubercle (OT) (Fig. 3b). DDAH2 immunoreactivity was also observed within the nucleus accumbens. In the striatum-like amygdalar nucleus, intense DDAH2 expression was found in the central amygdalar nuclei (CEA) (Fig. 3a, box 2), without staining in other nuclei such as the anterior or medial amygdalar nucleus. In the pallidum, DDAH2 staining was observed in the caudal part in the bed nuclei of the stria terminalis (BST) (Fig. 3c). There was no DDAH2 fluorescence signal in other regions of the pallidum. Within the cortical regions, DDAH2 immunoreactivity was restricted to layer II of the piriform cortex (Fig. 3a, box. 3) and layer II of the entorhinal area. Low DDAH2 expression was detected in the layer II of the auditory area, somatosensory, orbital, prelimbic, infralimbic and posterior parietal association areas in the cortex. Further, we observed region-specific DDAH2 expression in the HPF restricted to the pyramidal layer of CA1 while no signal was observed in CA2 and CA3 (Fig. 3a, box 1). Additionally, intense DDAH2 expression was observed in the subiculum and the presubiculum. Finally, within the cortical subplate, DDAH2 positive cells were restricted to the endopiriform nucleus.

Again, we compared our findings to the publicly available mRNA expression datasets (Allen Institute for Brain Science 2010; Linnarsson Lab 2020). *Ddah2* mRNA signals in the cortex, in olfactory areas and in the HPF, matched our protein expression data (refer to Table S4). However, there were differences in *Ddah2* mRNA distribution within the sub-regions of the HPF documented by the Allen Institute for Brain Science (Allen Institute for Brain Science 2010) and our data. According to the published data, *Ddah2* mRNA can be identified in CA1, CA2 and CA3, whereas Ddah2 protein signal in our study was restricted to CA1. Striatal protein expression was found but could not be compared in detail due to the limited sagittal data availability on the Allen Mouse Brain Atlas (Allen Institute for Brain Science 2010). Finally, *Ddah2* mRNA expression in the HPF, striatum, and hypothalamus was confirmed in our protein assessment in accordance with the known *Ddah2* mRNA distribution available at the Mouse Brain Atlas (Linnarsson Lab 2020).

### DDAH2 is Expressed Exclusively Within Neurons

**Fig. 4:**
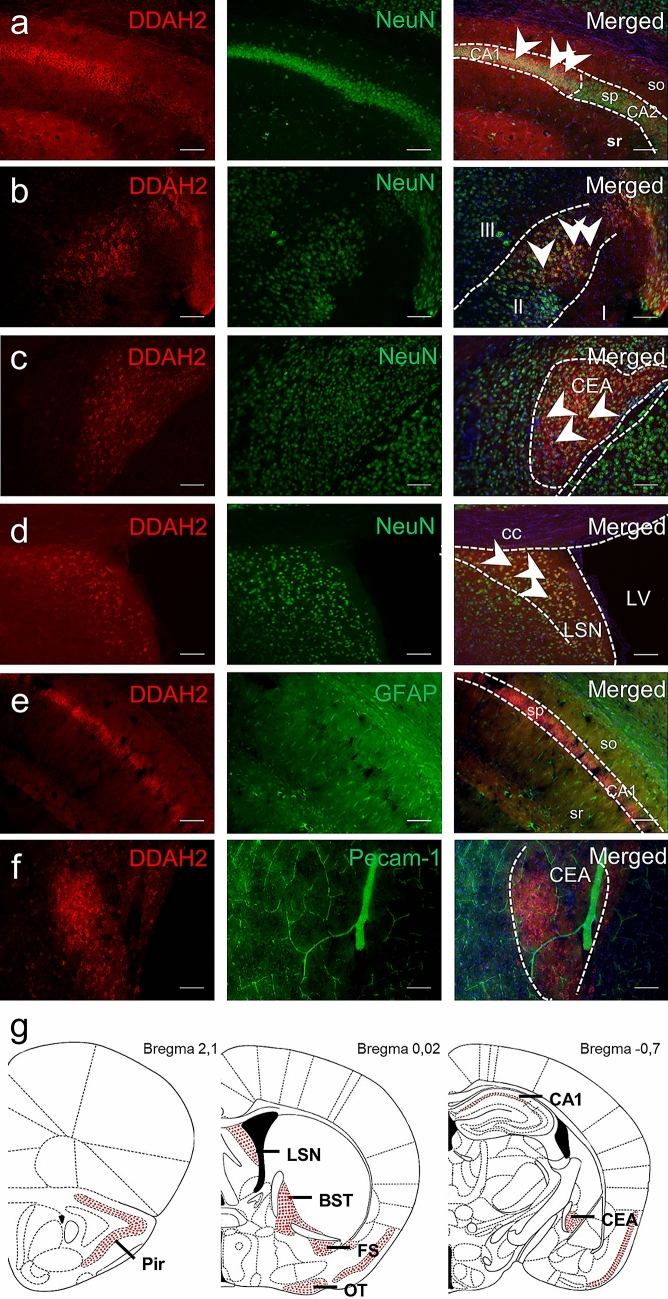
DDAH2 is expressed in neuronal cells. Mouse brain sections were co-stained with anti-DDAH2 (red, first column) and anti-neuronal marker (NeuN) antibodies (green, second column). **a** DDAH2/NeuN positive cells in the pyramidal layer (sp) of CA1, but not stratum oriens (so), stratum radialis (sr) and sp of CA2. **b** DDAH2/NeuN positive cells in layer II, but not layers I and III in piriform cortex. **c** Staining of the CEA shows DDAH2/NeuN co-localization **d** DDAH2/NeuN co-localization in lateral septal nucleus; cc—corpus callosum, LV – lateral ventricle. **e** No overlap of staining of DDAH2 and GFAP in HPF. **f** No overlap of staining of DDAH2 and PECAM-1 in CEA. **g** Overview scheme summarizing, DDAH2/NeuN positive cells (red dots) in piriform cortex (Pir); fundus of striatum (FS); bed nuclei of stria terminalis (BST); olfactory tubercle (OT); central amygdalar nucleus (CEA); Ammon’s horn (CA1); lateral septal nucleus (LSN). Nuclear staining in blue with DAPI. White arrows mark overlap. Scale bar is 100 µm

Based on the already obtained cell size and shape information, we performed a co-labeling analysis with the neuronal marker NeuN. We found a complete overlap of DDAH2 positive cells with NeuN in all structures (Fig. 4). DDAH2/NeuN expression was observed in CA1, while NeuN immunoreactivity continued in CA2 and CA3 (Fig. 4a). The neuronal origin of DDAH2 positive cells was also confirmed for layer II of the piriform cortex, while NeuN positive cells in both layers I and III did not express DDAH2 (Fig. 4b). The CEA showed specific DDAH2 and NeuN positive staining, which was not observed in other amygdalar nuclei (Fig. 4c). Intense DDAH2 and NeuN positive expression was seen in the caudal part of the LSN (Fig. 4d). For validation, we also performed an analysis of DDAH2 and GFAP double staining, which did not yield any co-labeling (Fig. 4e). Similarly, PECAM-1 positive cerebral endothelial cells did not display DDAH2 signal (Fig. 4f), despite strong signal in peripheral vascular beds (Supplementary Material, Fig. S5). Results of the immunocytochemical analysis on early postnatal primary cell culture of cortical and hippocampal origin confirmed the presence of DDAH2 in neurons; however, a minor fraction of DDAH2 positive cells also stained for GFAP (Fig. S4d–f). In summary, within the adult mouse brain, DDAH2 protein was found to be expressed exclusively in neuronal cells within a limited number of brain structures.

### DDAH1 and DDAH2 are Expressed by Different Cell Populations

**Fig. 5:**
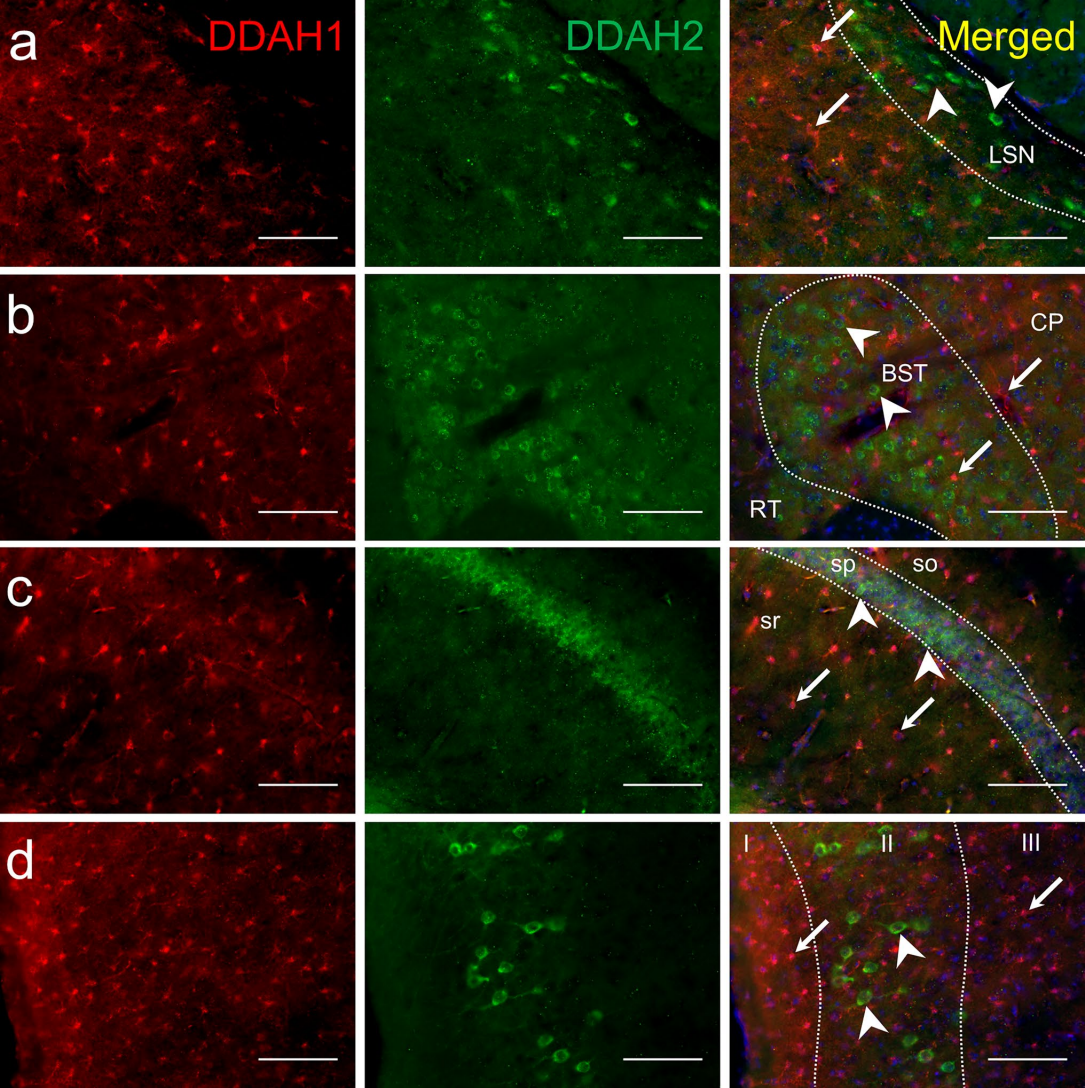
DDAH1 and DDAH2 do not overlap. Sagittal mouse brain sections were stained with anti-DDAH1 (red, first column) and anti-DDAH2 (green, second column). **a** DDAH1 and DDAH2 are both expressed in the lateral septal nucleus (LSN) but in different cells. Additionally, DDAH1 is found in caudoputamen (CP). **b** DDAH2 immunoreactivity was intense in bed nuclei of stria terminalis (BST). Here, DDAH1 cells were smaller and did not overlap with DDAH2. RT—reticular nucleus of the thalamus. **c** Neurons of pyramidal layer of CA1 of hippocampal formation (HPF) expressed DDAH2 but not DDAH1, which was present in glial cells of HPF. Sp—pyramidal layer; so—stratum oriens; sr—stratum radialis. **d** DDAH2 is expressed in large round cells in layer II of the entorhinal cortex, while DDAH1 positive cells were smaller and observed in all layers. White arrows and arrowheads indicate DDAH1 and DDAH2, respectively. Scale bar is 100 µm

Next, we performed co-labeling experiments of DDAH1 and DDAH2. As shown in Fig. 5, both DDAH1 and DDAH2 signals were observed in the HPF, cortex and striatum. However, DDAH1 and DDAH2 were consistently expressed by different cells. In the HPF, DDAH2 was present only in neurons of sp of CA1, whereas DDAH1 positive cells were observed in astrocytes in other layers (so, sr, slm) (Fig. 5c). In the striatum, DDAH2 was expressed by neurons in a few striatal structures such as LSN, whereas DDAH1 was broadly present in astrocytes (Fig. 5b). Additionally, DDAH2 and DDAH1 expression was observed in the BST (Fig. 5b) and entorhinal cortex but did not show cellular overlap Fig. 5d. In summary, our results suggest that DDAH1 and DDAH2 protein are detected in the same regions of the brain but always in a cell-type restricted manner.

### Identical Cell Types in Mouse and Human Brain Tissue Express DDAH1 and DDAH2

**Fig. 6:**
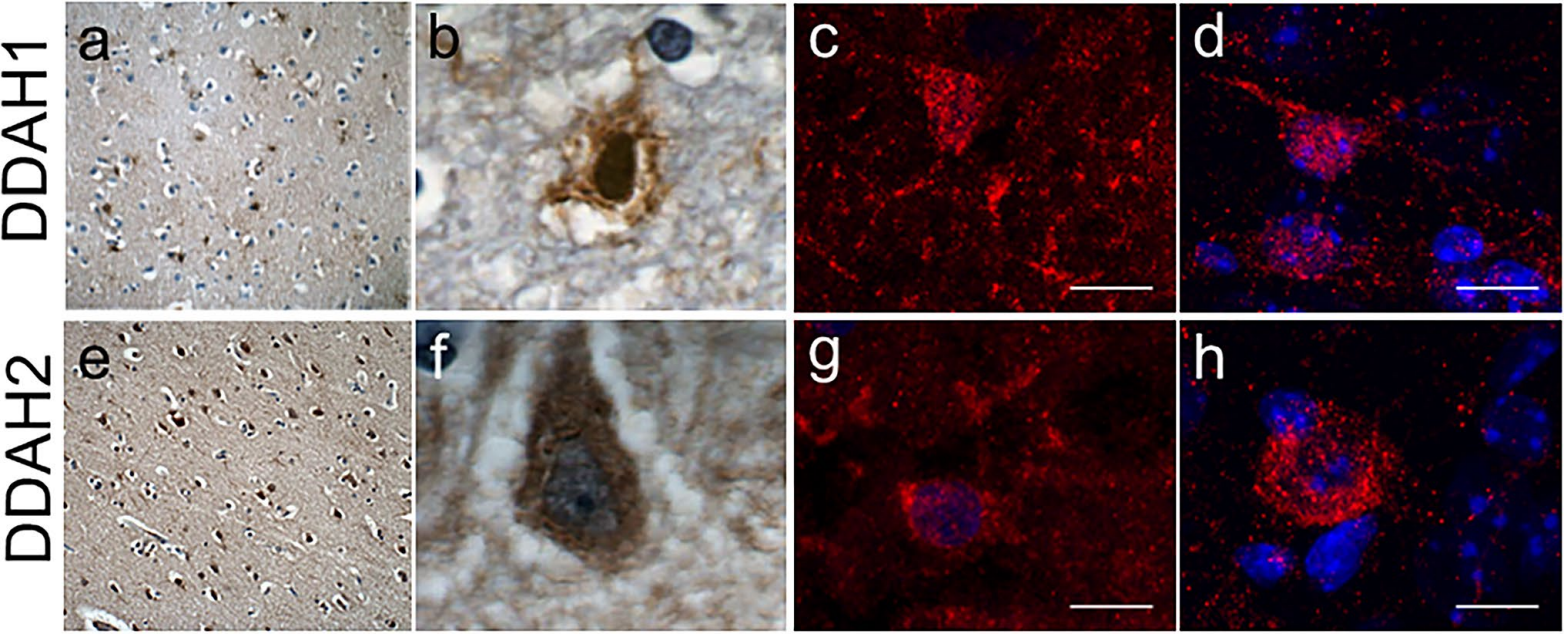
DDAH1 and DDAH2 are expressed by phenotypically similar cells in mouse and human brain tissue. Expression of DDAH1 and DDAH2 was compared in mouse and human brain tissue. Both DDAH1 (**a**) and DDAH2 **€** expression was observed in human medial frontal gyrus tissue with different morphological characteristics. While DDAH1 expression resembles astrocytes (**b**), strong DDAH2 expression is observed in neurons (**f**). Cortical DDAH1 positive cells have small soma and multiple processes in human (**b**) and murine samples (**c**, **d**). Amygdala DDAH2 immunoreactivity is observed in both human (**f**) and mouse (**g**, **h**). **a**, **e** 20 × , **b**, **f** 100 × , **c**, **d**, **g**, **h**. Scale bar is 10 µm

Finally, we performed a comparative analysis in human and murine tissue. We established staining of DDAH1 and DDAH2 on human post-mortem tissue to compare its distribution with our murine data (Fig. 6). At first, we performed DAB staining in tissue samples from the medial frontal gyrus, where both DDAH1 (Figs. 6a, b) and DDAH2 (Figs. 6e, f) signals were observed. DDAH1 positive cells appeared small (app. 15 µm^2^) and star-shaped with multiple processes reminiscent of astrocytes, whilst DDAH2 positive cells had neuronal features and an area of app. 19 µm^2^. Comparable findings are derived from DDAH1 expression in mouse (Fig. 6d) and human cortex (Fig. 6c) as well as DDAH2 staining on mouse (Fig. 6h) and human (Fig. 6g) amygdala. In summary, similar cell types express DDAH1 and DDAH2 in both human and mouse CNS tissue.

## Discussion

The main findings of our study are that: (1) DDAH1 is broadly distributed in the adult mouse brain; (2) DDAH1 is expressed in both neuronal and astrocyte cells as well as within the endothelia of the vascular structures; (3) DDAH2 is expressed exclusively in neuronal cells within a limited number of brain structures; (4) DDAH1 and DDAH2 can be expressed in the same brain region but not within the same cell; (5) The distribution pattern of DDAH1 and DDAH2 in the brain is similar in mice and humans.

Previous studies on murine DDAH1 protein expression have been sparse. In line with our histochemical results, a strong DDAH1 expression was observed in whole brain homogenate (Hu et al. 2011) and regional expression was reported for the hippocampus, dorsal root ganglia and spinal cord (DʼMello et al. 2015). Also, DDAH1 protein was detected in primary cell culture with cortical origin (Dowsett et al. 2015). Further support for our findings comes from databases reporting wide *Ddah1* mRNA distribution throughout the mouse brain and for distinct cell types (Linnarsson Lab 2020; Allen Institute for Brain Science 2010). Our findings confirmed the expression of DDAH1 in neurons and astrocytes but not in oligodendrocytes and low expression in endothelial cells. Nevertheless, the regional distribution of DDAH1 protein in the mouse brain is more limited compared to the database mRNA expression profile. The discrepancy may be explained by post-transcriptional and post-translational regulation in addition to differences in stability of both mRNA and protein (de Sousa Abreu et al. 2009). Another technical issue could be the use of antibodies. Although specificity was tested prior to application, sensitivity could be a limiting factor for the detection of low protein expression levels.

Likewise, an early study in human tissue samples from various organs showed strong *DDAH1* mRNA expression in the brain (Leiper et al. 1999), which was later confirmed by the Human Protein Atlas and the HGNC database (EMBL-EBI 2020; Human Protein Atlas 2020; Uhlén et al. 2015). Additionally, mRNA dot blot analysis using isoform-specific cDNA probes showed *DDAH1* expression in the amygdala, striatum, cerebellum, thalamus, hippocampus and various cortical areas (Tran et al. 2000). Besides acknowledging the wide mRNA expression in all brain regions tested, protein expression was found to be limited to the cerebral cortex, hippocampus, basal ganglia and cerebellum (Uhlén et al. 2015; Human Protein Atlas 2020). Prior studies in combination with our data, therefore, indicate similar expression profiles between human and murine datasets on both the mRNA and protein level.

Our study revealed a sparser distribution of DDAH2, compared to the widely represented DDAH1, in agreement with western blot analyses showing significantly lower expression of DDAH2 than DDAH1 in murine whole brain samples (Hu et al. 2011). Regional DDAH2 protein expression profile was limited to the cortex, hippocampus, striatum, and pallidum. This profile partially correlated with available mRNA expression data, which also included expression in the hypothalamus and cerebellum (Mouse ENCODE Consortium 2019; Stamatoyannopoulos et al. 2012; Linnarsson Lab 2020; Allen Institute for Brain Science 2010). Similarly, very low *DDAH2* mRNA distribution was detected in the human brain (Leiper et al. 1999) or found to be mostly restricted to the medulla and spinal cord (Tran et al. 2000). These findings were again confirmed by mRNA datasets, where *DDAH2* mRNA distribution in human brain tissue was barely present compared to the high level of expression observed in the other tissues, e.g., lung and fetal tissue (EMBL-EBI 2020; Human Protein Atlas 2020; Uhlén et al. 2015).

Additionally, we demonstrated for the first time that DDAH2 protein in the adult murine brain is found exclusively in neuronal cell types, which is in line with reported neuronal *Ddah2* mRNA expression (Allen Institute for Brain Science 2010; Linnarsson Lab 2020). The neuronal identity of DDAH2 positive cells was also confirmed in our immunocytochemical analysis on primary cell cultures of cortical and hippocampal origin. However, in primary cell culture, DDAH2 was additionally observed in astrocytes. The early postnatal origin of these samples and previous reports of high embryonic DDAH2 expression, which was found to decrease over development (Tran et al. 2000), may explain this finding. Of note, DDAH1 expression remains much more stable during development (Tran et al. 2000).

Studies demonstrating expression of DDAH2 in endothelial cells (Lin et al. 2019; Wang et al. 2007; Pullamsetti et al. 2005) and the hematopoietic lineage (Tran et al. 2000; Winkler et al. 2017; Lambden et al. 2015; Tomikawa et al. 2006) are in contrast with our observation of lacking signal in cerebral endothelial cells and microglia. It has been noted that gene expression patterns of the endothelium are highly heterogeneous and linked to specialized functional roles across vascular beds (Chi et al. 2003; Nolan et al. 2013). Such tissue-specific adaptations for brain endothelial cells include the instruction of neuronal differentiation and blood–brain-barrier formation (Jambusaria et al. 2020). Also, we do not exclude that either one of these cell types, endothelial cells and microglia, does express DDAH2 during non-adult stages. Besides the above-mentioned overall reduction in DDAH2 with age (Tran et al. 2000), a significant developmental decline between P14-P60 of *Ddah2* mRNA in microglia was observed (Zhang et al. 2014). Further, single-cell transcriptomics from mouse cortex and neurogenic niches (Zywitza et al. 2018; Zeisel et al. 2015) support low transcript levels in adults, possibly below our detection limits. Finally, upregulation of DDAH2 may occur in response to a stressor and during pathological conditions (Jambusaria et al. 2020; Gunawardana et al. 2021). This possibility is exemplified by the observed brain-specific up-regulation in endothelial transcripts in response to key mediators of tissue inflammation (Cleuren et al. 2019) and the DDAH2 increase in murine monocytes and human peripheral blood mononuclear cells in response to hypoxia (Lambden et al. 2016).

Together, our data indicate a distinct expression of DDAH isoforms in the brain. Such isolated patterns of expression of DDAH isoforms have also been observed in peripheral tissues. In the kidney, for example, DDAH1 is observed in the proximal tubules, while DDAH2 is expressed in the glomeruli, afferent arterioles, macula densa, and distal nephron, where each DDAH isoform has been found to regulate local ADMA concentrations in specific pathophysiologic processes (Onozato et al. 2008; Tomlinson et al. 2015). Observed regional and cellular specific expression profiles of DDAH1 and DDAH2 thus support our hypothesis of different functions or divergent influences on brain activity. Early studies already suggested that *DDAH1* is primarily co-expressed with *nNOS* in the brain and *DDAH2* is detected in tissues that also express *eNOS* and *iNOS* such as endothelial tissue and immune cells, respectively (Tran et al. 2000; Leiper et al. 1999; Suzuki-Yamamoto et al. 2012). Later it was shown that the DDAH1 expression patterns were completely different from nNOS expression patterns in rat and chicken during embryonic development (Mishima et al. 2004). Our study demonstrates that at least in the adult brain, it is DDAH2, rather than DDAH1, which is neuron specific. By itself, this does not imply nNOS co-expression and further analyses need to be performed for clarification. Additionally, it is possible that the different expression patterns of DDAH isoforms are caused by different regulatory mechanisms and maybe even different substrate preferences of these enzymes (Todd et al. 2001; Nguyen Ba et al. 2014). Indeed, while the major role of DDAH1 in the regulation of ADMA homeostasis is generally accepted, the relative contribution of DDAH2 towards ADMA clearance is still controversial (Wojciak-Stothard et al. 2007; Hasegawa et al. 2007; Hu et al. 2011; Wang et al. 2007). Even the data on whether DDAH2 is enzymatically active towards ADMA at all, are contrasting. On the one hand, global *Ddah2* knockout mice exhibit a significant increase in tissue ADMA concentrations (Lambden et al. 2015) and localized *Ddah2* upregulation in mice lung results in decreased tissue ADMA concentrations indicating that DDAH2 actively catabolizes ADMA (Aggarwal et al. 2014). Conversely, tissues of global DDAH1 deficient mice had no detectable enzymatic activity towards ADMA despite unchanged DDAH2 protein levels (Hu et al. 2011). In addition, ADMA-hydrolyzing activity was only observed in porcine tissues with high DDAH1 expression but absent in tissues with high DDAH2 expression lacking DDAH1, which would be consistent with DDAH2 being unable to hydrolyze ADMA (Altmann et al. 2012). As the role of DDAH2 in ADMA degradation remains debatable, further investigations of DDAH2 activity on ADMA are warranted to better understand the physiological and pathophysiological roles of this protein.

Alterations in DDAH activity in the human brain may contribute to the development of certain neuropsychiatric disorders. Changes in ADMA levels have been observed in patients with depression (Baranyi et al. 2015; Selley 2004; Braun et al. 2021), bipolar affective disorder (Sağlam Aykut et al. 2012; Braun et al. 2021), migraine (Greco et al. 2015), schizophrenia (Celik et al. 2011; Braun et al. 2021), Parkinson’s disease (Kirbas et al. 2016) and Alzheimer’s disease (Selley 2003). At the same time, there have been only limited advances in the diagnosis and treatment of these disorders in recent decades. Current therapies, mostly in the form of psychopharmacological agents, often lead to insufficient symptom relief and side effects. Thus, there is a need to develop new and targeted treatment strategies that specifically address the pathological processes from which symptoms arise. The described regional and cellular expression pattern of DDAH isoforms may facilitate the identification of specific pathophysiological roles played by these enzymes in the brain.

Further detailed investigations of DDAH isoform mutant mouse models may not only help to understand the functions of the DDAH/ADMA/NO pathway in the brain but also lead to the strategy of isoform-specific modulation of DDAH activity as a potential new therapeutic target for psychiatric disorders.

## Supplementary Information

Below is the link to the electronic supplementary material.

Supplementary file1 (PDF 810 kb)

Supplementary file2 (XLSX 12 kb)

Supplementary file3 (XLSX 11 kb)

Supplementary file4 (XLSX 18 kb)

Supplementary file5 (XLSX 14 kb)

## Data Availability

The data used and analyzed are available from the corresponding author on reasonable request.
