# Supplementary material for: Divergent Dimethylarginine Dimethylaminohydrolase Isoenzyme Expression in the Central Nervous System
Source: Cell Mol Neurobiol. 2021 May 20;42(7):2273–88. doi: 10.1007/s10571-021-01101-7 (PMC9418281; doi:10.1007/s10571-021-01101-7)
Supplement: Supplementary file 1 — Supplementary file1 (PDF 810 kb) [file 10571_2021_1101_MOESM1_ESM.pdf]

Electronic Supplementary File

Divergent dimethylarginine dimethylaminohydrolase isoenzyme expression in the central nervous system

Alena A. Kozlova[1,2*] ORCID: 0000-0002-0254-6264, Vinitha N. Ragavan[3,4*], Natalia Jarzebska[3,5], Iana V. Lukianova[1], Anastasia E. Bikmurzina[6] ORCID:0000-0002-4533-0834, Elena Rubets[3,6], Toshiko Suzuki-Yamamoto[7], Masumi Kimoto[7], Arduino A. Mangoni[4] ORCID:0000-0001-8699-1412, Raul R. Gainetdinov[2] ORCID:0000-0003-2951-6038, Norbert Weiss[3], Michael Bauer[1], Alexander G. Markov[6] ORCID:0000-0002-2867-044X, Roman N. Rodionov[3,4#] ORCID:0000-0002-7470-7776, Nadine Bernhardt[1#] ORCID:0000-0002-3188-8431

[1]Department of Psychiatry and Psychotherapy, University Hospital Carl Gustav Carus, Technische Universität Dresden, Dresden, Germany

[2]Institute of Translational Biomedicine and Saint-Petersburg University Hospital, Saint-Petersburg State University, Saint-Petersburg, Russia

[3]University Centre for Vascular Medicine and Department of Internal Medicine, Technische Universität Dresden, Dresden, Germany

[4]Department of Clinical Pharmacology, College of Medicine and Public Health, Flinders University and Flinders Medical Centre, Adelaide, Australia

[5]Department of Anesthesiology and Intensive Care Medicine, University Hospital Cart Gustav Carus, Technische Universität Dresden, Dresden, Germany

[6]Department of General Physiology, Saint-Petersburg State University, 199034 Saint-Petersburg, Russia,

[7]Department of Nutritional Science, Faculty of Health and Welfare Science, Okayama Prefectural University, Okayama, Japan

[*] - contributed equally

[#] - contributed equally

Corresponding author: Nadine Bernhardt, PhD; E-Mail: nadine.bernhardt@uniklinikum-dresden.de

Supplementary Material and methods

Cell Culture

Permission for use of umbilical cords for isolation of human umbilical vein endothelial cells (HUVECs) was given by the Ethikkommission of the Medical Faculty Carl Gustav Carus of TU Dresden. HUVEC isolation was performed according to the methods by Khedr *et al.* (2018) with some modifications. The HUVECs were grown in EBM-2, Endothelial Cell Growth Medium-2 (Lonza) supplemented with EGM-2, Endothelial SingleQuots Kit (Lonza) containing the following growth supplements: 10mL fetal bovine serum (FBS); 2mL human fibroblast growth factor-beta; 0.5mL vascular endothelial growth factor; 0.5mL R3-insulin like growth factor-1; 0.5mL ascorbic acid; 0.5mL human epidermal growth factor; 0.5mL Gentamicin/Amphotericin-B.

Cell lines used in this study were purchased from either American Type Culture Collection (ATCC) or Leibniz-Institute DSMZ – German Collection of Microorganisms and Cell Cultures (DSMZ). Rat parietal endoderm cell line (L2) (ATCC), human hepatocellular carcinoma cell line (HEPG2) (DSMZ), permanent human endothelium-derived cell line (EA.hy926) (ATCC), and human embryonic kidney 293 cell line (HEK293T) (DSMZ) were grown in Dulbecco's Modified Eagle's Medium with glutamine from Gibco supplemented with 10% FBS and 1% Penicillin/Streptomycin (Pen/Strep). Human prostate cancer cell line (PC3) (ATCC) was cultured in Ham's F-12K (Kaighn's) medium (Gibco) supplemented with 10% FBS and 1% Pen/Strep. Cells were grown as an even monolayer and dissociated during routine cell passaging with 0.05% Trypsin-ethylenediaminetetraacetic acid (EDTA) followed by neutralisation by the respective cell culture medium.

For the staining of primary cells, wildtype C57BL6/J P1-P3 pups of mixed sex were sacrificed. Cortical tissue was digested in 0.025% trypsin without separation of the hippocampus for 15 min at 37°C. 30% FBS-containing Neurobasal medium was applied and tissue filtered, centrifuged, cells dissolved and maintained in Neurobasal A medium containing antibiotics, 2% B27 (Invitrogen) and 0.5 mM glutamax (Invitrogen). The primary cell culture contained several cell types including but not limited to neurons and glial cells.

Animals and tissue collection

Global *Ddah1* (DDAH1$^{-/-}$) deficient C57Bl/6J mice were a kind gift from Professor Yingjie Chen, Cardiovascular Division, University of Minnesota Medical School, Minneapolis, USA. DDAH1$^{-/-}$ mice were bred from heterozygous parents (DDAH1$^{+/-}$) and genotyped with the following primer pairs; wild type allele forward 5′-AATCTGCACAGAAGGCCCTCAA-3′, reverse 5′-GGAGGATCCATTGTTACAAGCCCTTAACGC-3'; knock-out allele forward 5′-TGCAGGTCGAGGGACCTAATAACT-3′, reverse 5′-AACCACACTGCTAGATGAAGTTCC-3'. (Hu et al, 2011). The animals were housed in a 12-h light dark cycle (lights on at 06:00) with food and water *ad libitum*. All efforts were made to reduce animal suffering and number of animals used. Mice were deeply anesthetized with a mixture of 100 mg/kg ketamine and 10 mg/kg xylazine and perfused with phosphate buffered saline (PBS) during tissue harvest.

Heart tissues from *Ddah2* knockout in mice were a kind gift from Professor Renke Maas from the Institute for Experimental and Clinical Pharmacology and Toxicology, Friedrich-Alexander-University Erlangen-Nürnberg (FAU), Erlangen, Germany, who purchased the mice from Taconic.

Immunocytochemistry

Cells grown on coverslips were fixed with 4 % paraformaldehyde (PFA) for 15 min and permeabilized in 0.1 % (v/v) Triton X-100 in PBS for 5 min at room temperature. Cells were blocked in 3 % bovine serum albumin (BSA) in PBS at 37°C for 1 hr followed by overnight incubation at 4°C in primary antibody solution (3 % BSA in PBS) (refer to Table 1 and Table S1-S2). Cells were washed 3 x 10 min in PBS and later incubated with fluorescence conjugated secondary antibody (805-167-008; Jackson Immuno Research, USA) (3 % BSA in PBS) for 1 hr at room temperature. Cells were washed 3 x 5 min in PBS and later stained with DAPI (1:1000 in PBS) for 5 min at room temperature. Cells were washed 3 x 5 min in PBS and mounted in Fluoromount-G (SouthernBiotech, USA). Images were capture using KEYENCE BZ-X800 fluorescence microscope.

Primary cells were used for staining 10 days after preparation. Fixation was performed with 4 % PFA for 15 min, followed by 3 x 10 min washing steps in PBS and permeabilization in 0.3 % (v/v) Triton X-100 in PBS for 10 min. Blocking was performed in 10 % either goat or donkey serum in PBS depending on the species of the secondary antibody. Primary antibodies were diluted in PBS (refer to Table 1 and Table S1) and samples incubated over night at 4°C on a shaker. Afterwards cells were 3 x 10 min washed in PBS, incubated with secondary antibody (refer to Table 1) for 2 hrs at room temperature, washed again 3 x 15 min in PBS and counterstained with DAPI for 10 min. Followed by 1 x 15 min washing in PBS, cells were mounted on SuperFrost plus glass cover slides with Mowiol 4-88. Samples were visualized using Zeiss Observer with ApoTome, images processed with ImageJ software to enhance the signal intensity.

Western Blot

Tissue samples collected from mice were homogenized, (2 x 20 seconds at 6500 rpm) and sonicated (3 x 10 seconds burst, 60 % amplitude (AMP), 4°C) in cold RIPA buffer (0.15M NaCl, 0.05M Tris, pH 8.0, 1 % NP-40, 0.5 % DOC, 0.1 % SDS) containing a protease inhibitor (Mini-complete Protease Inhibitor Cocktail Tablets; Roche Diagnostics-Applied Science, Mannheim, Germany). Cells were harvested by scratching and incubated in cold RIPA buffer containing a protease inhibitor for 30 min at 4°C before sonication (3 x 10 seconds burst, 40 % AMP, 4°C). Post sonication, lysed tissue and cell samples were centrifuged (14 000 x g, 5 min, 4°C) and the supernatant was measured for protein concentration using Pierce BCA Protein Assay Kit (ThermoFisher Scientific) according to manufacturer's instruction. For immunoblot analysis, 20 µg of cell or tissue lysates were prepared and diluted with Laemmli buffer (0.25M Tris-HCl, 8 % SDS, 40 % glycerol, 0.2 mg/mL bromophenol blue, 20 % β-mercaptoethanol). Samples were denatured at 95°C for 10 min and separated by sodium dodecyl sulfate polyacrylamide gel electrophoresis under reducing conditions on 10 % polyacrylamide gels (20 min at 100V followed by 1 hr at 150V). The samples were later transferred to polyvinylidene difluoride membranes (PVDF Western Blotting Membrane, Roche, Mannheim, Germany) using a tank blotting system from Bio-Rad (Munich, Germany) at 100V for 1 hr at 4°C. Membranes were blocked in blocking buffer TBS (50mM Tris-HCl, pH 7.4, 150mM NaCl) and 3 % milk powder for 1 hr followed by overnight incubation at 4°C in primary antibody solution TBST (TBS, 0.2 % Tween20) with 2 % milk powder containing primary antibodies (refer to Table 1 and Table S1-S2). Membranes were washed 3 x 10 min in TBST and incubated with horseradish peroxidase conjugated secondary antibody (111-035-144; 205-035-108; Jackson Immuno Research, USA) for 1 hr at room temperature. Membranes were washed 3 x 15 min in TBST before the immunoreactive bands were visualised using Roche "Lumi-Light Western Blotting Substrate" (Roche, Germany) on PeqLab Fusion Fx7 Imaging System (Peqlab, Germany).

Supplementary Figures

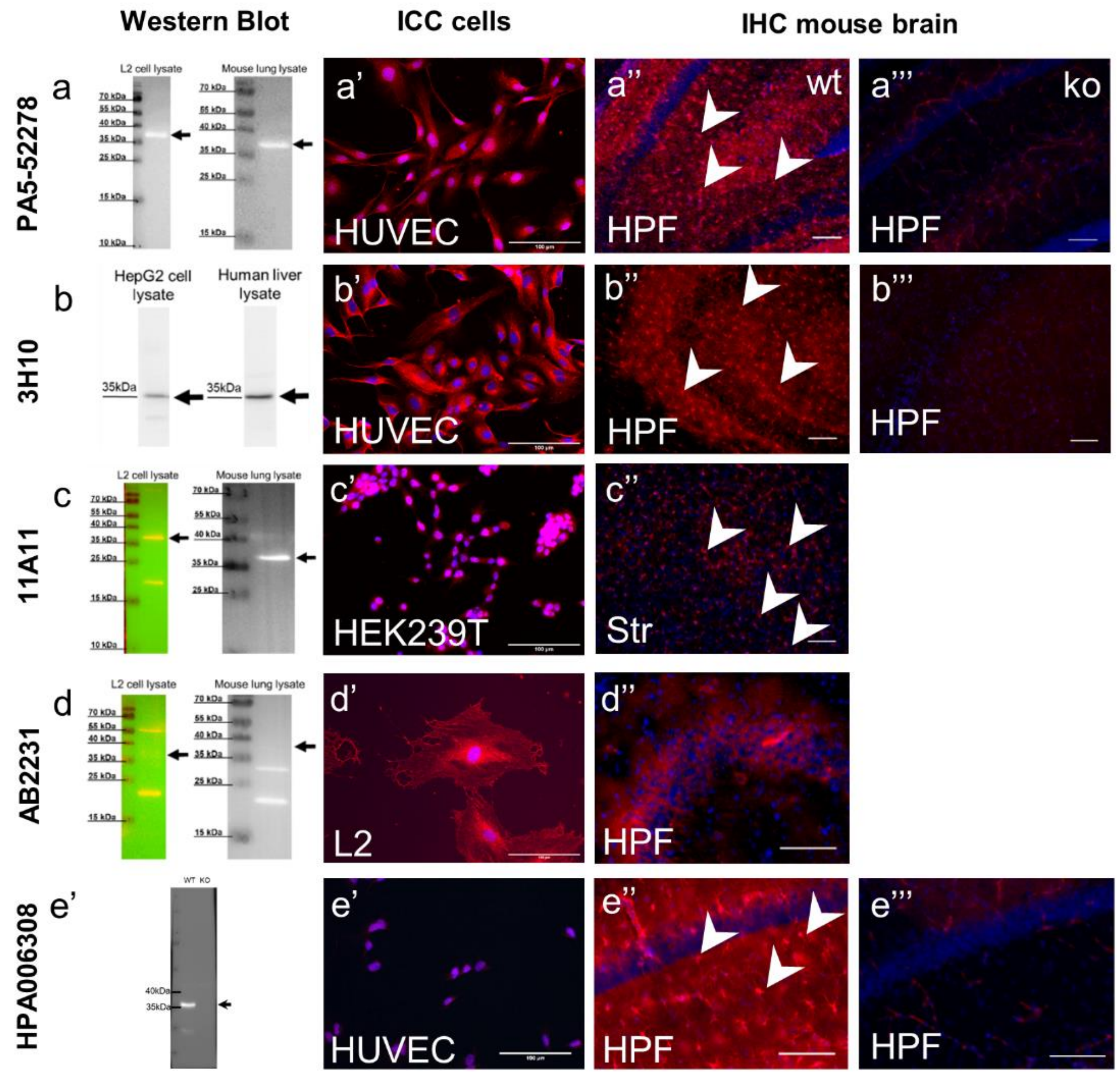

Figure S1. Immunohistochemical (IHC) and immunocytochemical (ICC) analyses and western blot (WB) validation of available anti-DDAH1 antibodies. Staining was performed with PA5-52275 (ThermoFisher, USA), ab2231 (Abcam, UK), HPA006308 (MERCK, USA) and clones 11A11 and 3H10 antibodies. Analyses were conducted with different cell lines, and mouse brain tissue sections. WB analyses were performed in rat pulmonary epithelial cell line (L2) and mouse lung lysate with PA5-52275 (a), 11A11 (c) and ab2231 (d). Clone 3H10 anti-DDAH1 was used for WB analyses on human liver cancer cell (HEPG2) lysate and human liver lysate. ICC analyses were conducted on human umbilical vein endothelial cells (HUVEC; a', b', e'), human embryonic kidney 293 (HEK293T; c') and L2 (d') cell lines. Validation of HPA006308 was performed on lung lysates from *Ddah1* knockout (KO) and wildtype (WT) mice. Band size app. 37 kDa. Following staining of hippocampal formation (HPF) in WT and *Ddah1* KO mouse brain sections, PA5-52278 (a''; a'''), 3H10 (b'', b''') and HPA006308 (e'', e'''), were used in the main study providing strong specific staining. Scale bar – 100 µm. Arrows in tissue staining experiments indicate DDAH1 positive cells

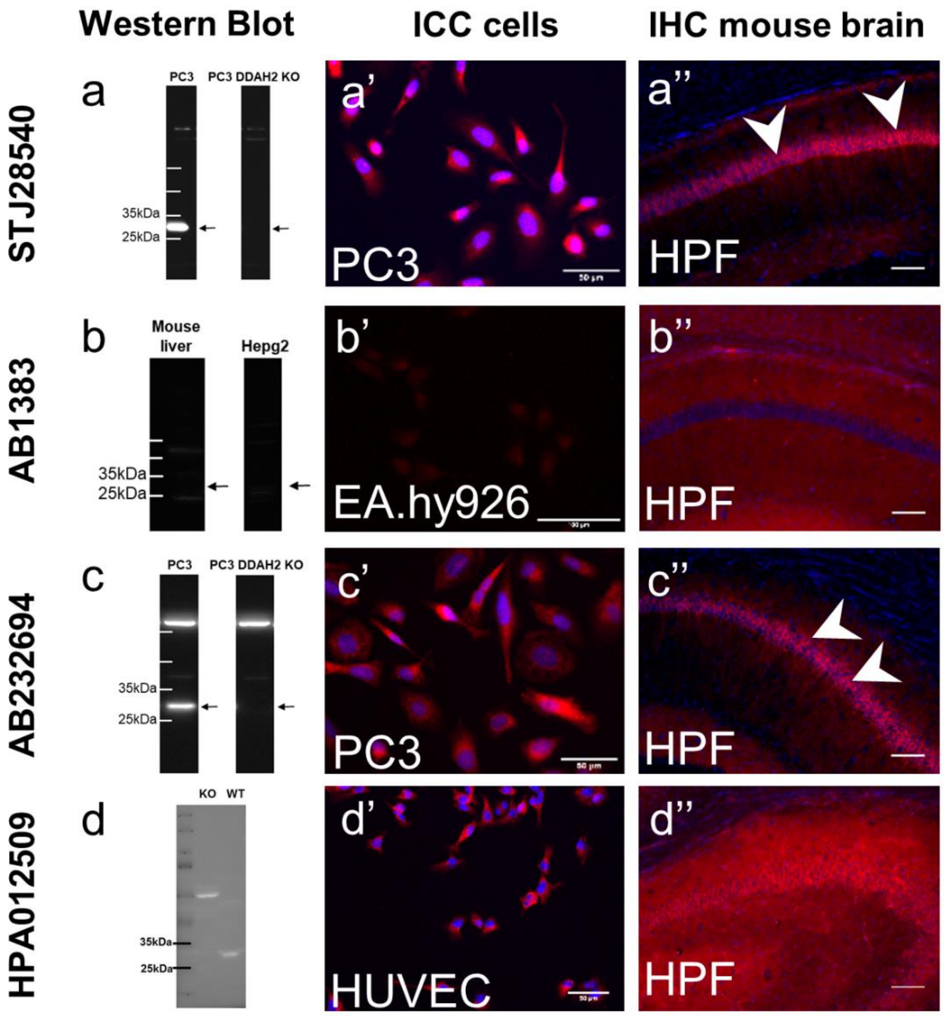

Figure S2. Immunohistochemical (IHC) and immunocytological (ICC) analyses and western blot (WB) validation of commercially available anti-DDAH2 antibodies. Staining was performed with STJ28540 (St. John's Lab, UK), ab1383, ab232694 (Abcam, UK) and HPA012509 (MERCK, USA) antibodies. Validation was conducted with various cell lines, and mouse brain tissue sections. Specific and well stained bands in western blots analyses were observed in wildtype (WT) and *Ddah2* knockout (KO) human prostate cancer cell line (PC3) with STJ28540 (a) and ab232694 (c). Analyses of mouse liver and human liver cancer (Hepg2) lysates with ab1383 (b) showed no specific signal. Additionally, ab1383 performed weak staining on permanent human endothelium-derived cell line (EA.hy926; b') and mouse brain tissue (b''). ab1383 was used for human tissue staining in main study, however, currently, this antibody is not available by Abcam. Validation of HPA012509 was established with *Ddah2* KO and wildtype (WT) mouse heart tissue lysate. Western blot analyses showed specific band in WT without a band in *Ddah2* KO tissue (d). Band size ~30 kDa. IHC and ICC analyses confirmed western blot's results (a', c', d'). Following immunohistochemistry in hippocampal formation (HPF) mouse brain tissue, STJ28540 (a'') and ab232694 (c'') were used in main research due to their strong specific staining. Scale bar – 100 µm. White arrows in tissue staining experiments indicate DDAH2 positive cells.

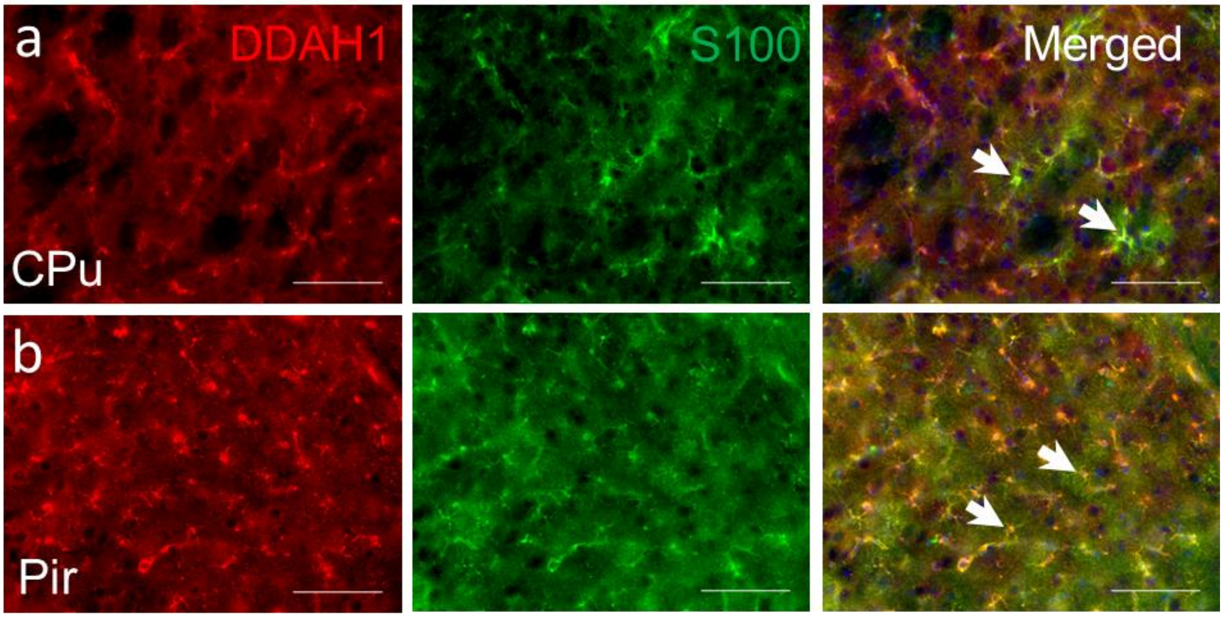

Figure S3. Immunohistochemical analyses of DDAH1 and astrocyte marker S100ßeta showing overlap a) in caudoputamen (CPu) and b) piriform cortex (Pir). Scale bar – 100 µm. Arrows indicate double positive cells

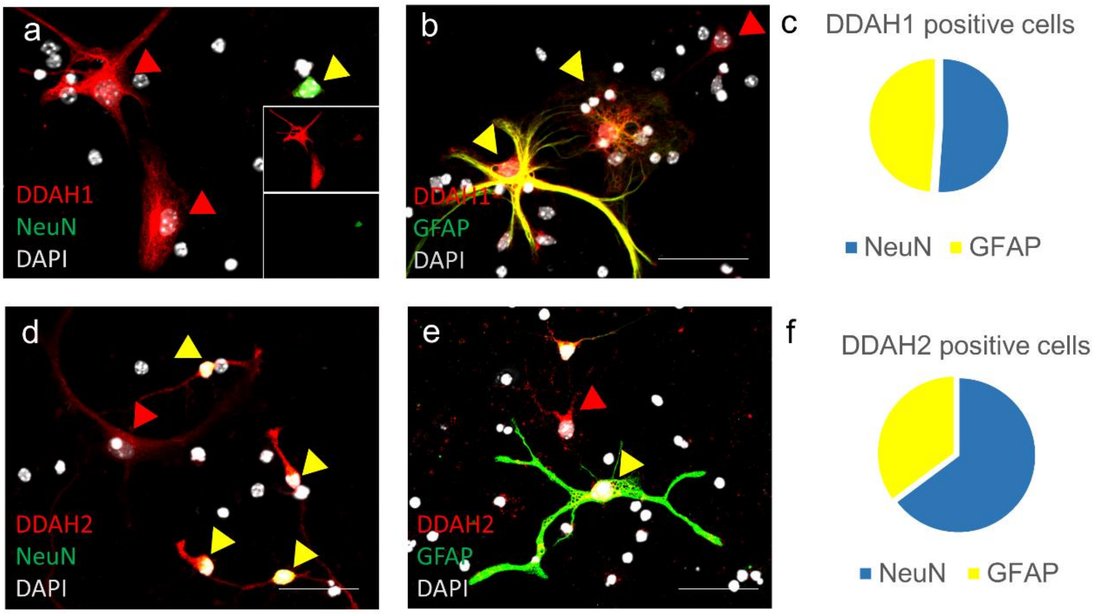

Figure S4. Immunocytochemical analysis of cell specific expression of DDAH1 and DDAH2 distribution in primary cortical and hippocampal murine cell culture. Double staining with NeuN as neuronal marker (a, d) and GFAP as marker for astrocytes (b, e) was performed on primary cells. Red arrows indicate cells positive for solely DDAH1, green arrows represent cells positive for the cell specific marker, yellow arrows point at double-positive cells. DDAH1 expression in astrocytes was considered strong and distributed across the whole cells, including cell processes with weaker signal in the nucleus (a, b). DDAH1 expression in neurons was generally weaker than in

astrocytes (data not shown). DDAH1 was expressed in equal ratio in both neurons and astrocytes (c). DDAH2 was predominantly found in neurons (f) but staining in astrocytes was observed with weak signal intensity (e). Scale bar - 50 µm

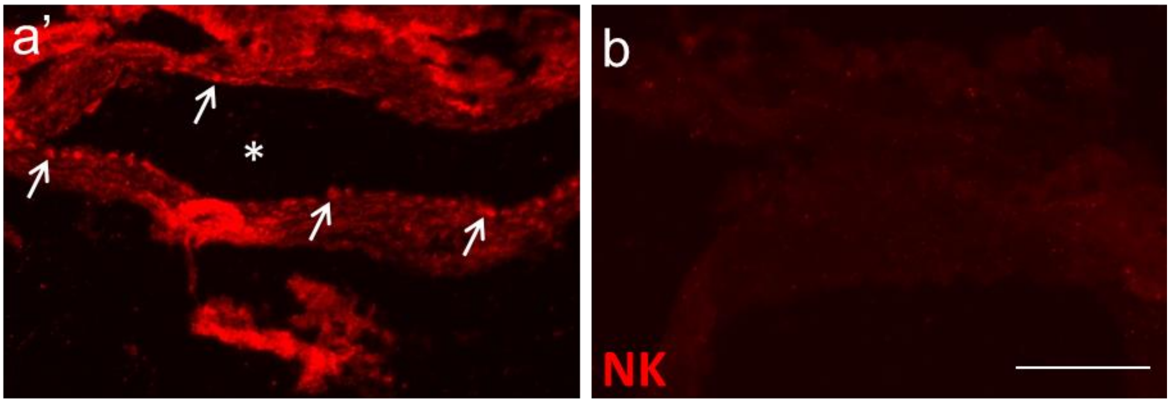

Figure S5. Immunohistochemical analyses of DDAH2 in mouse aorta using ab232694. Sections (5µm) stained with (a) anti-DDAH2 antibody (red) and (b) negative control (NK) only secondary antibody Alexa 568. Specific staining against DDAH2 in the close to the lumen (*) area marked with arrows. confirmed by. Scale bar – 200 µm.

Supplementary References

Hu X, Atzler D, Xu X, Zhang P, Guo H, Lu Z, Fassett J, Schwedhelm E, Boger RH, Bache RJ, et al (2011) Dimethylarginine dimethylaminohydrolase-1 is the critical enzyme for degrading the cardiovascular risk factor asymmetrical dimethylarginine. Arterioscler Thromb Vasc Biol 31: 1540-6. https://doi.org/10.1161/atvbaha.110.222638

Khedr S, Deussen A, Kopaliani I, Zatschler B, Martin M (2018) Effects of tryptophan-containing peptides on angiotensin-converting enzyme activity and vessel tone ex vivo and in vivo. Eur J Nutr 57: 907-915. https://doi.org/10.1007/s00394-016-1374-y
